# Acute gross and histopathologic changes after ischaemic ventricular tachycardia ablation

**DOI:** 10.1093/ehjcr/ytaf605

**Published:** 2025-11-22

**Authors:** Hyo-Jeong Ahn, Eun Na Kim, Jiwon Koh, Eue-Keun Choi

**Affiliations:** Division of Cardiology, Department of Internal Medicine, Seoul National University College of Medicine and Seoul National University Hospital, 101 Daehak-ro, Jongno-gu, Seoul 03080, Republic of Korea; Department of Pathology, Seoul National University College of Medicine and Seoul National University Hospital, 101 Daehak-ro, Jongno-gu, Seoul 03080, Republic of Korea; Department of Pathology, Seoul National University College of Medicine and Seoul National University Hospital, 101 Daehak-ro, Jongno-gu, Seoul 03080, Republic of Korea; Division of Cardiology, Department of Internal Medicine, Seoul National University College of Medicine and Seoul National University Hospital, 101 Daehak-ro, Jongno-gu, Seoul 03080, Republic of Korea

## Case description

A 60-year-old man underwent emergent coronary artery bypass grafting due to triple-vessel coronary artery disease after cardiac arrest for acute myocardial infarction (MI). He consequently developed severe ischaemic cardiomyopathy (ICM) and received catheter ablation a month after MI for incessant ventricular tachycardia (VT) under extracorporeal membrane oxygenation (ECMO) support, following failed weaning due to haemodynamic instability. Radiofrequency (RF) energy was delivered to delayed fragmented potentials in the basal-inferior left ventricle (LV) using an irrigated catheter (ThermoCool SmartTouch SF, Biosense Webster). Extensive substrate modification was inevitable due to sustained VT with varying morphology and wobbling cycle length. A total of 162 ablation points were applied over 58.7 min (40 W, irrigation flow 15 mL/min), with a median contact force of 10.4 g [interquartile range (IQR) 8.3–14.1], a median ablation index of 423.8 (IQR 322.6–558.1), and a baseline impedance of 80.1 ± 2.5 ohms, with an average impedance drop of 4.1 ± 2.1%. The relatively low baseline impedance and an attenuated impedance drop during ablation might be attributable to the patient’s pathological status—namely, underlying severe ICM as well as the concomitant ECMO support. Due to recurrent VT in severe ICM, the patient underwent orthotopic heart transplantation 6 days after VT ablation.

**Figure 1:**
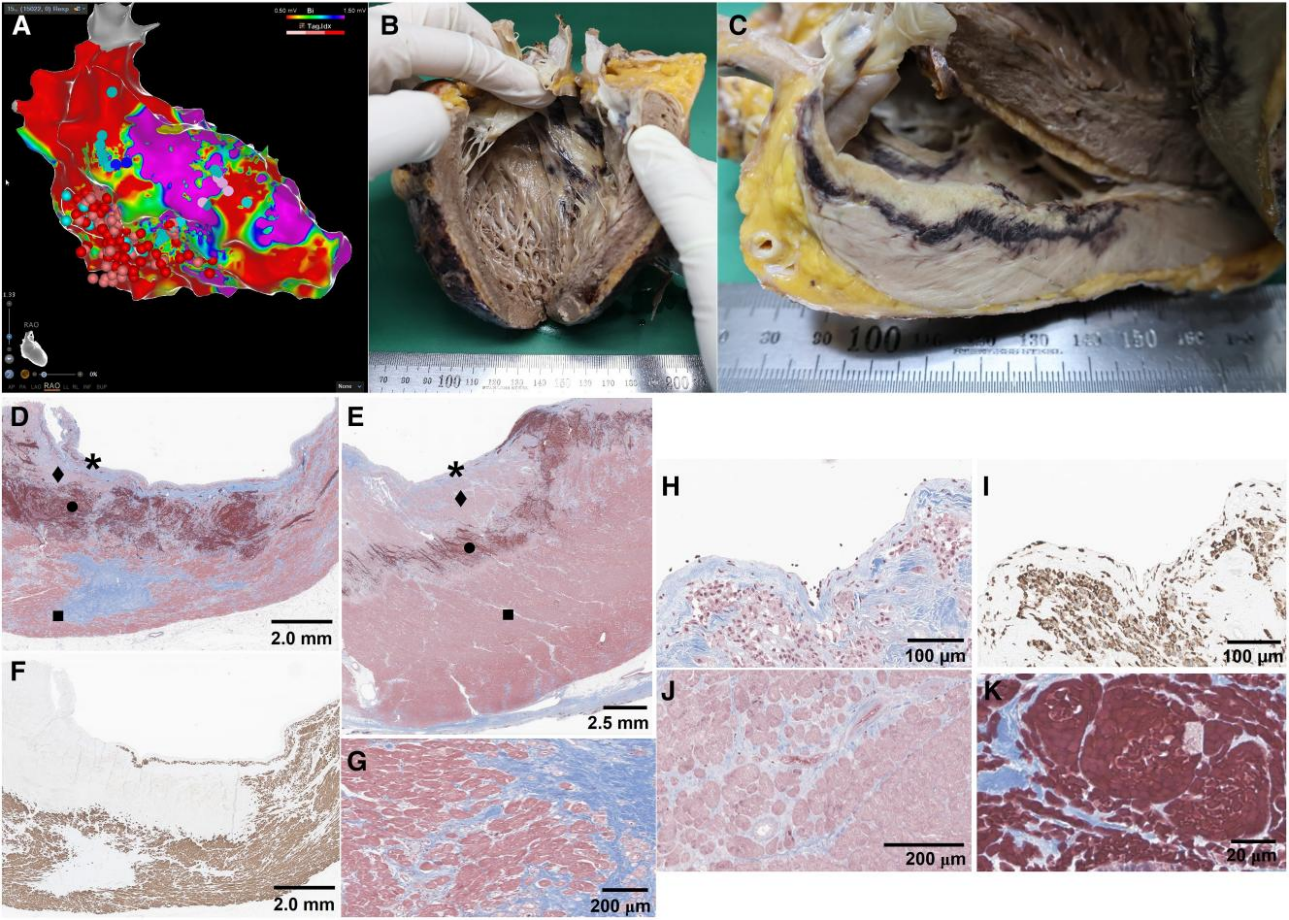
(*A*) Voltage mapping of left ventricle and ablation points; (*B* and *C*) gross anatomy of the radiofrequency ablation lesion for ventricular tachycardia; (*D* and *E*) Masson’s trichrome stain of a representative section of ablation lesion; (*F*) desmin stain of (*D*) showing damaged cardiomyocytes by ablation and infarct; (*G*) ■ portion of (*D*) and (*E*) showing heterogeneous ventricular tachycardia substrate of a mixture of viable and ischaemically damaged cardiomyocytes; (*H*) * portion of (*D*) and *E*) showing neutrophils and histiocytes infiltration in endomyocardium; (*I*) CD68 stain of (*H*) demonstrating mixed inflammatory infiltration composed of neutrophils and histiocytes; (*J*) ♦ portion of (*D*) and (*E*) showing interstitial oedema and contraction band necrosis; (*K*) ● portion of (*D*) and (*E*) showing coagulative necrosis and diffuse interstitial haemorrhagic rim.

The explanted heart showed a round necrotic area with a haemorrhagic rim in the basal-inferior LV wall and a one-third myocardial thickness ablation lesion (*Figure 1A–C*). Histopathology revealed three layers of ablation lesion on a mixture of viable and ischaemically damaged cardiomyocytes: (i) neutrophils and histiocytes infiltration in endomyocardium, (ii) interstitial oedema and contraction band necrosis beneath it, and (iii) coagulative necrosis and diffuse interstitial haemorrhage at deep edge of the lesion (*Figure 1D–K*).

Although steam pop lesions have been reported following VT ablation, detailed characterization of acute VT ablation lesions, particularly in human myocardium, remains limited.^1,2^ These gross and histopathological images, obtained 6 days after RF ablation, illustrate a heterogeneous ischaemic VT substrate that had just entered the chronic phase of MI remodelling.^3^ Also, they illustrate a characteristic three-layered structure of an acute RF ablation lesion while highlighting the challenge of achieving transmurality. We hypothesize that the deep haemorrhagic border may impede further lesion formation by altering tissue impedance and the lesion’s irreversible injury that may contribute to delayed VT suppression. In the era of pulsed field ablation (PFA), RF remains the mainstay for VT ablation. This case illustrates the complexities of RF lesion formation and serves as a useful reference for comparison with lesion created by PFA.

## Data Availability

The data underlying this article will be shared on reasonable request to the corresponding author.

## Notes

**Consent:** The patient declared consent for scientific research, according to the COPE guidelines.

**Funding:** None declared.
